# Isolation and Molecular Characterization of Swine Parainfluenza Virus 5 from Piglets Co-Infected with PEDV

**DOI:** 10.3390/vetsci12070676

**Published:** 2025-07-18

**Authors:** Yuling Ma, Xinxin Chen, Mengyao Ma, Xiaolong Gao, Ruoqi Song, Yue Yi, Ying Wang, Sheng Niu, Yujun Zhao, Wenxia Tian, Jianle Ren, Fang Yan

**Affiliations:** 1 College of Veterinary Medicine, Shanxi Agricultural University, Jinzhong 030801, China; mayuling@solarbio.com (Y.M.); jjdwswx@126.com (X.C.); mamengyao03@163.com (M.M.); songruoqi0711@163.com (R.S.); yueyi_2025@163.com (Y.Y.); wangying@sxau.edu.cn (Y.W.); niusheng@sxau.edu.cn (S.N.); tgzhaoyujun@163.com (Y.Z.); wenxiatian@126.com (W.T.); 2 Beijing Solarbio Science & Technology Co., Ltd., Beijing 101102, China; 3 Beijing Animal Disease Prevention and Control Center, Beijing 102629, China; veterinary89@126.com

**Keywords:** parainfluenza virus 5, swine, isolation, phylogenetic analysis, cross-species transmission

## Abstract

Parainfluenza virus 5 (PIV5) is recognized as a pathogen capable of infecting diverse animal species and humans, posing a potential threat to public health. In this study, the PIV5 strain SC2024 was isolated from the PEDV-positive intestinal tissue of piglets. Using next-generation sequencing (NGS), we determined the full genome length of the virus to be 15,246 nucleotides (nt). Phylogenetic analysis showed that SC2024 grouped into the same branch (Lineage 2.2) as most of the previously reported swine-origin PIV5 strains in China. Additionally, it exhibited genetic similarity with isolates from various hosts, such as tigers, pangolins, lesser pandas, and ticks. Overall, these findings expand our comprehension of PIV5 epidemiology in swine populations, and underscore the importance of further research and surveillance.

## Simple Summary

Parainfluenza virus 5 (PIV5), a member of the *Orthorubulavirus* genus within the *Paramyxoviridae* family, infects diverse mammalian species, including humans. In this study, our primary objective was to isolate porcine epidemic diarrhea virus (PEDV) from intestinal tissue samples of infected piglets. Unexpectedly, we successfully isolated the PIV5 strain (SC2024) from diarrhea piglets. Genetic analysis revealed that SC2024 clustered within the same branch as most previously reported swine-derived PIV5 strains in China, and shared some genetic characteristics with strains isolated from diverse hosts, including tigers, pangolins, lesser pandas, and ticks. Collectively, these findings enhance our comprehension of PIV5 epidemiology in swine populations and highlight its cross-species transmission potential.

## 1. Introduction

Parainfluenza virus 5 (PIV5), now reclassified as *Orthorubulavirus mammalis*, is a negative-sense, single-stranded RNA virus belonging to the genus *Orthorubulavirus* of the family *Paramyxoviridae* [1,2,3,4]. The PIV5 genome comprises 15,246 nucleotides in length and encodes eight viral proteins: nucleoprotein (NP), V protein, phosphoprotein (P), matrix protein (M), fusion protein (F), small hydrophobic protein (SH), hemagglutinin–neuraminidase protein (HN), and large protein (L) [5,6]. Notably, the V and P proteins are generated from the V/P gene; these two proteins share an overlapping genomic region and are generated through a specific RNA editing mechanism [7,8,9].

Originally, PIV5 was identified in primary monkey kidney cells in 1954 [10]. Since then, it has been widely detected across diverse host species, including humans, dogs, pigs, cats, calves, equines, geese, pangolins, and lesser pandas, with varying clinical manifestations reported [2,11,12,13,14,15,16,17,18]. For instance, in calves, PIV5 is associated with severe respiratory illness and neurological disorders, contributing to high morbidity rates [11,19]. In dogs, PIV5 is recognized as a common pathogen, resulting in moderate respiratory illness or neurological dysfunction; however, severe clinical signs may develop when co-infected with other respiratory viruses or bacteria [20,21,22,23]. Of note, accumulating evidence has indicated that it has significant potential for cross-species transmission [2,14,24,25,26]. Supporting this, the swine PIV5 strain GX2020, the equine PIV5 strain XJ033, and the human PIV5 reference strains were clustered within the same clade and exhibited a close genetic relationship with the human-derived strain AGS [13,15]. Cross-species transmission has also been documented in coyotes, ferrets, and rodents [27,28].

In pigs, the first PIV5 isolate designated, SER, was identified in the lung tissue of a sow co-infected with porcine reproductive and respiratory syndrome virus (PRRSV) [29]. Subsequently, more PIV5 strains were isolated from pigs with respiratory and diarrheal symptoms in China and South Korea [30,31,32,33]. In this study, the primary objective was to isolate pandemic PEDV strains from the intestinal tissues of PEDV-infected piglets; however, we unexpectedly isolated a PIV5 strain. Of note, a recent study demonstrated that PIV5 had been associated with diarrhea in pigs [32]. Therefore, our isolation of PIV5 from diarrheic piglets might provide further evidence suggesting a potential link between PIV5 and porcine diarrhea. To better understand PIV5 epidemiology in swine, we performed genetic variation and evolutionary analyses on this isolate. Collectively, this study enhances our understanding of PIV5′s epidemiological status in swine populations.

## 2. Materials and Methods

### 2.1. Cells and Clinical Samples

Vero cells were cultured in Dulbecco’s modified Eagle’s medium (DMEM) (Gibco, Grand Island, New York, USA) supplemented with 10% fetal bovine serum (FBS) (Gibco, Grand Island, New York, USA) at 37 °C in a humidified 5% CO_2_ incubator, as previously described [34]. Three intestinal tissues were collected from piglets exhibiting clinical diarrhea on a pig farm in Yibin, China.

### 2.2. Virus Isolation and Identification

Intestinal tissues were first screened for major enteric viruses, including PEDV, transmissible gastroenteritis virus (TGEV), porcine rotavirus (PoRV), and porcine deltacoronavirus (PDCoV) using reverse-transcription PCR (RT-PCR). Briefly, intestinal tissues were homogenized in DMEM under sterile conditions. Total RNA was extracted from tissue homogenates using a TIANamp Virus DNA/RNA Kit (Tiangen, Beijing, China), followed by cDNA synthesis with the PrimeScript™ II 1st Strand cDNA Synthesis Kit (Takara, Beijing, China), according to the manufacturers’ instructions. Viral nucleic acids were detected via PCR using virus-specific primers (Table S1) under the following conditions: 95 °C for 3 min; 30 cycles of 95 °C for 15 s, 55 °C for 30 s, and 72 °C for 30 s; and a final extension of 72 °C for 7 min.

For virus isolation, the tissue homogenates were centrifuged at 12,000× g for 10 min and filtered through 0.22 μm membranes to remove bacteria. Subsequently, Vero cells were inoculated with the filtered supernatant containing 10 µg/mL trypsin–EDTA(Gibco, Grand Island, New York, USA). Following a 1 h incubation, the inoculum was removed and replaced with DMEM supplemented with 10 µg/mL trypsin-EDTA [35,36]. The cells were incubated at 37 °C and examined daily for a cytopathic effect (CPE). Upon CPE observation, supernatants from infected cells were collected for viral nucleic acid extraction using the TIANamp Virus DNA/RNA Kit (Tiangen Beijing, China), followed by pathogen identification and genome sequencing via next-generation sequencing (NGS; Illumina platform, San Diego, CA, USA). Subsequently, the presence of PIV5 was further confirmed by RT-PCR using PIV5 NP gene-specific primers (Table S1).

### 2.3. Phylogenetic Analysis and Sequence Alignment

To examine the evolutionary relationship between the isolate PIV5 SC2024 and reference strains, the representative PIV5 sequences were obtained from GenBank database, and selected based on geographic distribution and host species. Phylogenetic analysis was performed based on the NP, F, and HN gene sequences of the isolate, along with its complete genome sequence, using MEGA 7 (Mega Limited, Auckland, New Zealand) with the neighbor-joining algorithm, 1000 bootstrap replicates, and the Kimura 2-parameter substitution model, as previously described [20,30,34,37]. For genetic characterization, the nucleotide sequences and deduced amino acid sequences of the NP, F, and HN genes were aligned and analyzed using Geneious Prime software (version 2022.2, Biomatters, Auckland, New Zealand) with the Clustal Omega program.

## 3. Results

### 3.1. Isolation and Identification of PIV5

**Figure 1:**
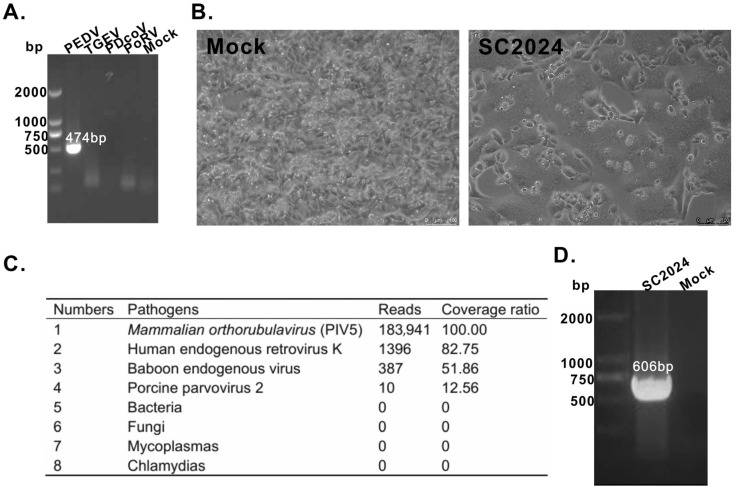
Isolation and identification of PIV5. (**A**) PCR verification of virus infection in intestinal tissues. (**B**) CPE of PIV5 on Vero cells. (**C**) Identification of PIV5 via NGS. (**D**) RT-PCR verification of PIV5.

To identify viral infections in intestinal tissues, RT-PCR was performed using virus-specific primers for PEDV, TGEV, PoRV, and PDCoV (Table S1). As shown in Figure 1A, only PEDV tested positive among all tissue samples. Subsequently, the tissue homogenates were inoculated onto Vero cells. And the CPE was observed after three passages in one of the tissue samples (Figure 1B). Notably, RT-PCR failed to detect the PEDV genome. Then, NGS was performed to identify the CPE causative pathogen using an Illumina platform. As shown in Figure 1C, NGS generated 183,941 reads with a 100% coverage ratio for PIV5. To further validate the NGS results, RT-PCR confirmed that the supernatants of virus-infected cells were positive for PIV5 (Figure 1D). Then, the newly isolated virus was designated SC2024. Following sequence assembly and annotation, the complete genome of SC2024 was determined to be 15,246 nt in length, containing a 5′ UTR, a 3′ UTR, and six open reading frames (ORFs) that code for the NP, V/P, M, F, NH, and L proteins. Then, the complete genome sequence was submitted in GenBank under accession number PV395591.1.

### 3.2. Phylogenetic Analysis

**Figure 2:**
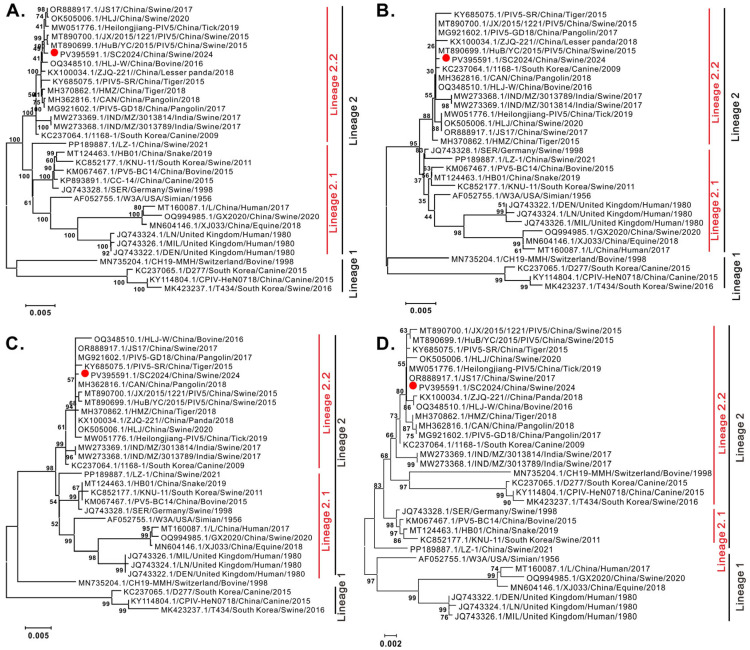
Phylogenetic analysis of the PIV5 strain SC2024. Phylogenetic trees were constructed using MEGA 7 software with the neighbor joining algorithm, 1000 bootstrap replicates, and the Kimura 2-parameter substitution model. The isolate PIV5 strain SC2024 is marked with red point. (**A**) Phylogenetic analysis of PIV5 based on whole-genome sequences. (**B**) Phylogenetic analysis of PIV5 based on NP sequences. (**C**) Phylogenetic analysis of PIV5 based on F sequences. (**D**) Phylogenetic analysis of PIV5 based on HN sequences.

To elucidate the genetic relationships between PIV5 SC2024 and the reference strains, phylogenetic trees were constructed using the neighbor joining method via MEGA 7.0 software, based on the NP, F, and HN genes, and complete genomic sequences. As shown in Figure 2, all phylogenetic trees revealed that PIV5 strains divided into two distinct lineages (Lineage 1 and Lineage 2), with lineage 2 further diverging into two sub-lineages (Lineage 2.1 and Lineage 2.2). Significantly, strain SC2024 clustered within the tertiary sub-lineage 2.2 alongside most Chinese swine-derived strains, which also included strains isolated from pangolins, tigers, lesser pandas, and ticks.

### 3.3. Sequence Identity and Alignment Analysis

Comparison of complete genomic sequences revealed that SC2024 shared 96.9–99.9% nucleotide identity with other PIV5 strains. Additionally, the NP, F, and HN genes displayed nucleotide identities of 96.8–99.9%, 95.8–99.9%, and 96.6–100% with those of other PIV5 strains, respectively. At the amino acid level, the encoded NP, F, and HN proteins showed sequence homologies of 97.6–99.8%, 94.9–99.8%, and 97.3–100% compared to their counterparts in other PIV5 strains (Table 1). Among pig-derived strains, the SC2024 demonstrated a higher degree of similarity to Chinese strains, particularly the JS17 strain. Among strains from other hosts, SC2024 showed greater similarity to strains 1168-1 (Canine), HLJ-W (Bovine), CAN (Pangolin), PIV5-GD18 (Pangolin), HMZ (Tiger), ZJQ-221 (Lesser panda), and Heilongjiang-PIV5 (Tick). Notably, the HN protein of SC2024 displayed 100% identity with that of the tick-derived strain Heilongjiang-PIV5.

**Figure 3:**
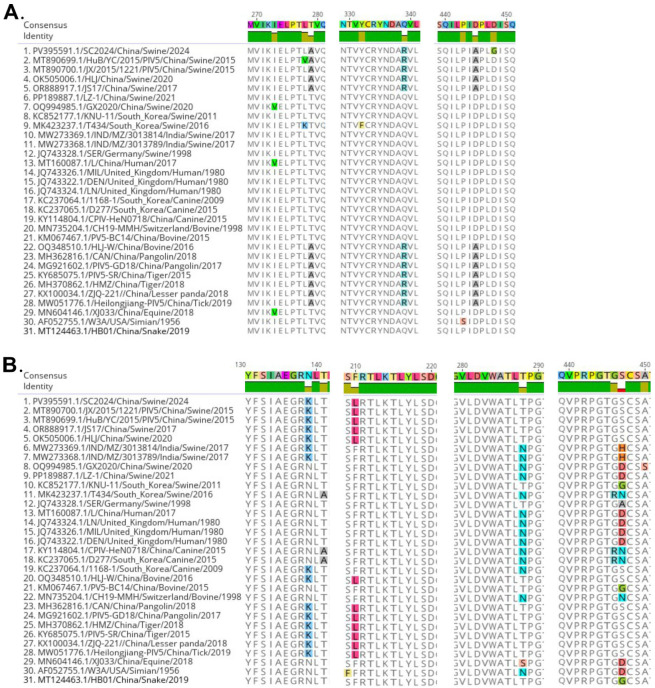
Multiple-sequence alignment of F and HN protein sequences among the PIV5 strains. (**A**) Alignment of F protein sequences, (**B**) Alignment of HN protein sequences.

The glycoproteins F and HN, which are critical importance for viral attachment, membrane fusion, and host cell entry, were subjected to further analysis. As shown in Figure 3A, compared with the reference strains, the F protein of SC2024 harbored a novel amino acid mutation (D448G). Notably, three amino acid sites (A279, R339, A445) in the F protein were conserved across SC2024 and strains HLJ-W, CAN, PIV5-GD18, PIV5-SR, HMZ, ZJQ-221, and Heilongjiang-PIV5, which originated from diverse hosts. Correspondingly, four amino acid positions (K139, L210, T288, S447) in the HN protein of SC2024 showed complete amino acid identity with those in strains CAN, PIV5-GD18, PIV5-SR, HMZ, ZJQ-221, and Heilongjiang-PIV5 (Figure 3B). These findings suggest that the F and HN proteins may serve as key determinants in PIV5 cross-species transmission.

## 4. Discussion

In this study, PIV5 strains were isolated from diverse host species [2,11,12,13,14,15,16,17,18]. This virus is associated with mild respiratory and diarrheal symptoms in pigs [32,38]. In this study, our original aim was to isolate PEDV from intestinal tissues of PEDV-affected piglets. However, we unexpectedly isolated the PIV5 strain SC2024. To better understand PIV5 epidemiology in swine, we performed genetic characterization analyses on this isolate. Our findings revealed the following: (i) phylogenetic analysis indicated that strain SC2024 was clustered within the same branch as most previously isolated swine-derived strains in China; and (ii) in terms of sequence homology and specific amino acid variation in HN and F proteins, SC2024 shared genetic similarities with the strains isolated from other host species, including tigers, pangolins, lesser pandas, and ticks.

We provide further evidence to reinforce the association between PIV5 infection and diarrhea in pigs. In swine populations, more PIV5 strains have been identified in pigs in both China and South Korea [15,30,38]. Recent serological surveys demonstrated a 75.7% PIV5 positivity rate in Chinese pig farms, indicating a high prevalence across swine facilities in China [24]. Our team successfully isolated the PIV5 strain SC2024 from the intestinal tissues of piglets co-infected with PEDV. Initially, our research aim was to isolate the prevalent strains of PEDV. However, during the virus passage process, PEDV unexpectedly disappeared, while PIV5 successfully propagated in the cells. We hypothesize that factors such as viral competition or tissue sample processing may have contributed to the failure to isolate PEDV. Similarly, a parallel study has reported the isolation of PIV5 from PEDV-affected pigs, in which PEDV also disappeared during viral passage [31]. In another study, animal trials revealed that PIV5 strain LZ-1 infection induces diarrhea in piglets, albeit with moderate pathogenicity [32]. When it comes to the pathogenicity of PIV5 in pigs, similar pathogenicity assessments of the KNU-11 strain have demonstrated either non-pathogenic or mildly pathogenic effects in pigs [38]. Given its low virulence or non-virulence nature, PIV5 represents a promising candidate for developing viral-vectored vaccines [3,39]. To date, several PIV5-based vaccine candidates have proven effective in protecting against viral infections in multiple animal models [40,41,42,43,44]. For instance, a recent report showed that PIV5-based SARS-CoV-2 vaccine could induce protective and long-lasting immunity in nonhuman primates [45,46]. However, current research on PIV5 vector vaccines primarily focuses on human and zoonotic pathogens, such as influenza viruses, coronaviruses, rabies virus (RABV), vaccinia virus (VACV), respiratory syncytial virus (RSV), HIV, or bacteria [3,39,40,41,42,43,44,45,46,47,48]. In contrast, studies targeting porcine pathogens remain significantly more limited. Thus, future experiments are required to construct a PIV5 reverse genetic system, which will pave the way for the development of vector vaccines against PRRSV and PEDV.

Our studies also provide a glimpse into the evidence of the potential cross-species transmission of PIV-5 through genetic analyses. Genome-wide phylogenetic analysis demonstrated that SC2024, together with the majority of pig-derived PIV5 strains in China, clusters within Lineage 2.2 (Figure 2A). Notably, this sub-lineage also encompasses strains originating from diverse host species, including tigers, pangolins, lesser pandas, and ticks. Similar results were observed in the phylogenetic analyses of the NP, F, and HN genes (Figure 2B–D), reinforcing the evolutionary relatedness across species. Parallel findings were reported for strain YN01 in a separate study [16]. The viral proteins F and HN facilitate viral entry and are critical targets for neutralizing antibodies in parainfluenza viruses [5,6,49]. NP is the most abundant protein in the virion, and it is believed to mediate RNA-dependent RNA polymerase activity [5,6,49]. Given their functional importance and genetic characteristics, the NP, F, and HN genes are frequently used as the basis for phylogenetic analyses across parainfluenza viruses, including Newcastle disease virus (NDV) [50] and parainfluenza virus 1–5 [33,37,51,52,53]. Supporting this cross-species potential, Ibrahim et al. also isolated a PIV5 strain from diarrheic piglets that was phylogenetically closely related to strains from lesser pandas and pigs in China. This strain had a broad host range, which was capable of infecting cell lines derived from multiple species, including pigs, humans, monkeys, cattle, dogs, cats, rabbits, hamsters, and mice [24]. Furthermore, our study also indicated that SC2024 shared some identical amino acid variation sites with the PIV5 strains of other species, including tigers, pangolins, lesser pandas, and ticks (Figure 3). Together, these results suggest that the PIV5 strain SC2024 possesses cross-species transmission potential.

Pigs are frequently recognized as potential intermediate hosts for viruses with zoonotic potential, such as influenza A virus (IAV) and Nipah virus (NiV), posing significant public health concerns [54,55,56,57]. In the context of PIV5, a previous study identified a human-like strain (GX2020) in pigs coinfected with PRRSV, highlighting the virus’s cross-species transmission risk [15]. Earlier research has linked six amino acid residues (22, 49, 57, 254, 378, and 460) in the HN gene to human-specific adaptations [53]. Notably, these sites in SC2024 did not exhibit mutations associated with human tropism. Furthermore, the receptor-binding domain of the PIV5 HN protein has been identified as ^186^QDHVS^190^, with cleavage sites located at E390 and Y523 [58]. Residues 37, 342, 437, and 457 are also recognized as being linked to viral entry into host cells and the generation of neutralizing antibodies [59]. In our study, these amino acids were conserved in SC2024 and other pig-derived strains within Lineage 2.2. The strain LZ-1 is considered to induce diarrhea in piglets through pathogenicity experiments [32]. However, phylogenetic tree analysis reveals that LZ-1 and SC2024 reside in distinct sub-branches (Figure 2). Amino acid sequence alignment further identified variations at positions 279, 339, and 445 in the F protein, and 139, 210, 288, and 447 in the HN protein between these two strains (Figure 3). This raises the question of whether these mutations affect viral pathogenicity, which remains unknown and requires further investigation in the future.

In summary, this study successfully isolated a novel PIV5 strain (SC2024) from PEDV-coinfected piglets, and performed a comprehensive genetic variation analysis. These findings deepen our understanding of PIV5 epidemiology in swine populations, and highlight the urgency of enhancing viral surveillance for PIV5 in pigs or other animal species.

## Figures and Tables

**Table 1:** Comparison of the nucleotide (nt) and amino acid (aa) of SC2024 with the reference PIV5 strains.

No.	Strain	GenBank No.	Year	Host	Country	SC2024						
						WGS	NP			F	HN	
						nt (%)	nt (%)	aa (%)	nt (%)	aa (%)	nt (%)	aa (%)
	GX2020	OQ994985.1	2020	Swine	China	97.2	97.6	98.2	97.5	97.3	96.6	96.5
	JX	MT890700.1	2015	Swine	China	99.8	99.9	99.8	99.8	99.5	99.8	100
	HuB/YC	MT890699.1	2015	Swine	China	99.8	99.8	99.8	99.8	99.5	99.9	100
	JS17	OR888917.1	2017	Swine	China	99.9	99.9	99.6	99.9	99.8	100	100
	LZ-1	PP189887.1	2021	Swine	China	98.8	99.2	98.6	99.1	98.4	98.9	97.9
	HLJ	OK505006.1	2020	Swine	China	99.9	99.8	99.6	99.9	99.8	99.8	99.8
	KNU-11	KC852177.1	2011	Swine	South Korea	98.7	99.1	98.4	99.0	98.2	99.1	98.4
	T434	MK423237.1	2016	Swine	South Korea	96.6	96.6	97.6	95.8	94.9	97.5	97.3
	IND/MZ/3013814	MW273369.1	2017	Swine	India	99.6	99.7	99.4	99.6	98.9	99.5	98.9
	IND/MZ/3013789	MW273368.1	2017	Swine	India	99.6	99.7	99.4	99.6	98.9	99.5	98.9
	SER	JQ743328.1	1998	Swine	Germany	99.0	99.5	99.4	99.1	98.4	99.4	98.9
	L	MT160087.1	2017	Human	China	97.3	97.8	98.4	97.3	96.9	96.8	96.5
	LN	JQ743324.1	1980	Human	United Kingdom	98.0	98.1	98.6	98.1	97.8	97.8	97.9
	MIL	JQ743326.1	1980	Human	United Kingdom	98.0	98.0	98.6	98.2	98.0	97.8	97.9
	DEN	JQ743322.1	1980	Human	United Kingdom	98.1	98.2	99.0	98.2	98.0	97.9	98.1
	CPIV-HeN0718	KY114804.1	2015	Canine	China	96.9	96.8	98.2	96.6	96.0	97.7	97.5
	1168-1	KC237064.1	2009	Canine	South Korea	99.8	99.9	98.8	99.7	99.1	99.9	99.6
	D277	KC237065.1	2015	Canine	South Korea	97.1	97.0	98.0	96.6	96.2	97.8	97.5
	HLJ-W	OQ348510.1	2016	Bovine	China	99.8	99.9	99.8	99.6	98.4	99.8	99.8
	PV5-BC14	KM067467.1	2015	Bovine	China	98.9	99.3	99.2	99.1	98.4	99.3	98.6
	CH19-MMH	MN735204.1	1998	Bovine	Switzerland	97.7	98.1	97.6	97.7	97.3	97.8	97.5
	CAN	MH362816.1	2018	Pangolin	China	99.8	99.9	99.8	99.9	99.8	99.8	99.6
	PIV5-GD18	MG921602.1	2017	Pangolin	China	99.8	99.9	99.8	99.9	99.8	99.8	99.6
	PIV5-SR	KY685075.1	2015	Tiger	China	99.7	99.7	99.2	99.8	99.8	99.9	99.8
	HMZ	MH370862.1	2015	Tiger	China	99.8	99.8	99.6	99.9	99.6	99.9	99.8
	XJ033	MN604146.1	2018	Equine	China	97.4	98.0	98.6	97.6	97.5	96.9	96.6
	W3A	AF052755.1	1956	Simian	USA	98.7	99.2	99.2	98.6	98.1	98.4	97.5
	ZJQ-221	KX100034.1	2018	Lesser panda	China	99.8	99.7	99.6	99.9	99.8	99.8	99.8
	HB01	MT124463.1	2019	Snake	China	98.9	99.5	99.4	99.1	98.4	99.2	98.6
	Heilongjiang-PIV5	MW051776.1	2019	Tick	China	99.8	99.8	99.6	99.8	99.5	100	100

## Data Availability

All data generated or analyzed during this study are included in this published article.

## Supplementary Materials

The following supporting information can be downloaded at: https://www.mdpi.com/article/10.3390/vetsci12070676/s1, Table S1: Primers for amplification of the genes in this study.
