# Supplementary material for: Isolation and Molecular Characterization of Swine Parainfluenza Virus 5 from Piglets Co-Infected with PEDV
Source: Vet Sci. 2025 Jul 18;12(7):676. doi: 10.3390/vetsci12070676 (PMC12299374; doi:10.3390/vetsci12070676)
Supplement: Supplementary file 1 [file vetsci-12-00676-s001.zip › vetsci-3726158-supplementary.pdf]

Table S1. Primers for amplification of the genes in this study

Virus	Primer Sequences (5′-3′)	Genes	Sizes
PEDV	F: AGCTTTCAGGTCAATTGGG	M	474 bp
	R: TTAGACTAAATGAAGCACTTTCTC		
TGEV	F: TCGCAATAATAGTAATGACCTTTAT	S	480 bp
	R: TTAAACCACCAAAGGTCTACAA		
PDcoV	F: GGCAAATTATTGTTTTCATTGCGATCATATGGGCGC	M	625 bp
	R: CTTATACAGGCGAGCGTCACCGGCCTTTGAAG		
PoRV	F: ATGTATGGTATTGAATATACCACAGTT	VP7	785 bp
	R: TGTATWAYWGCTACRTTYTCYCTTGGTCC		
PIV5	F: CAAGATCAGAGTGAGGAAGGTACA	NP	606 bp
	R: GATTATTCTTCGAGCCTCCGGTTG		
